# Endoscopic submucosal dissection for hypopharyngeal cancer simultaneous with immunotherapy for renal cell carcinoma

**DOI:** 10.1002/deo2.70009

**Published:** 2024-09-23

**Authors:** Tatsuro Hirao, Daisuke Kikuchi, Satoshi Yamashita, Hidehiko Takeda, Kenta Watanabe, Yuji Miura, Shu Hoteya

**Affiliations:** ^1^ Department of Gastroenterology Toranomon Hospital Tokyo Japan; ^2^ Department of Gastroenterology Toranomon Hospital (Branch) Kanagawa Japan; ^3^ Department of Otorhinolaryngology Toranomon Hospital Tokyo Japan; ^4^ Department of Medical Oncology Toranomon Hospital Tokyo Japan

**Keywords:** double cancer, endoscopic submucosal dissection, hypopharyngeal cancer, less invasive therapy, local control

## Abstract

We report a case of a man who was diagnosed with superficial hypopharyngeal cancer and recurrence of renal cell carcinoma in the duodenum, liver, and gluteus medius muscle simultaneously. He underwent endoscopic submucosal dissection for hypopharyngeal cancer in parallel with systemic immunotherapy for recurrent renal cell carcinoma, resulting in completely overcoming both malignancies. Endoscopic submucosal dissection is less invasive and can be performed in a shorter duration for treating superficial hypopharyngeal cancer compared with other treatment options, such as radiation therapy, chemotherapy, and surgery. Additionally, endoscopic submucosal carcinoma is adequately effective in controlling local lesions and has a satisfactorily good prognosis.

## INTRODUCTION

Endoscopic submucosal dissection (ESD) for early‐stage pharyngeal cancer is a curative procedure and less invasive than other treatments. This advantage enables local control of pharyngeal cancer in cases of concurrent malignancies, thereby conserving physical strength to treat the accompanying malignancies. However, few reports of ESD have been reported in such a situation. Here, we report a case of a man who was diagnosed with simultaneous hypopharyngeal cancer and recurrent renal cell carcinoma (RCC). He underwent ESD for hypopharyngeal cancer in parallel with immunotherapy for RCC, causing successful treatment of both malignancies and disease‐free survival to this day.

## CASE REPORT

**FIGURE 1:**
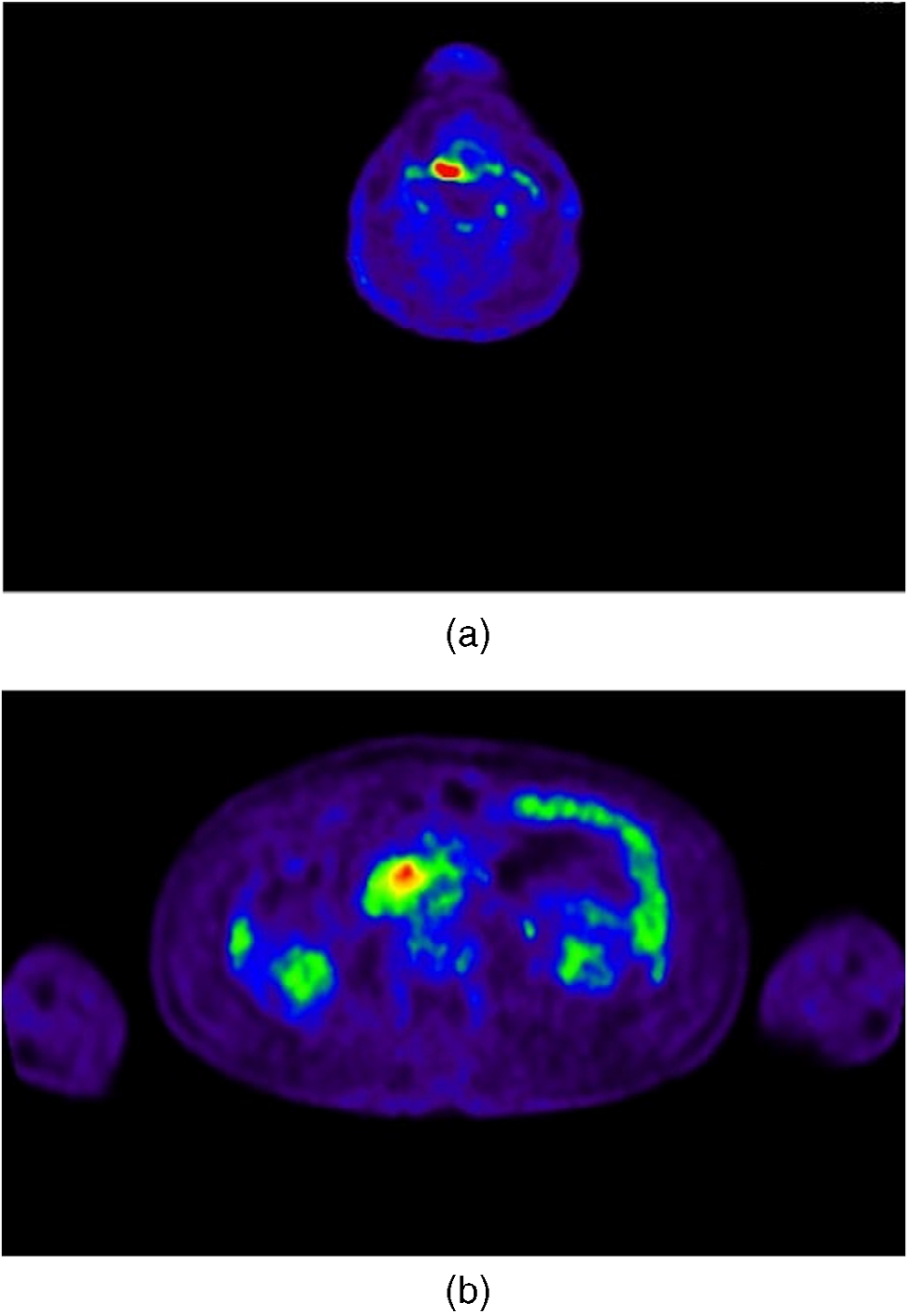
(a) Fluorodeoxyglucose hyperaccumulation in the right neck (SUVmax = 9.29). (b) FDG hyperaccumulation in the duodenum (SUVmax = 4.92).

**FIGURE 2:**
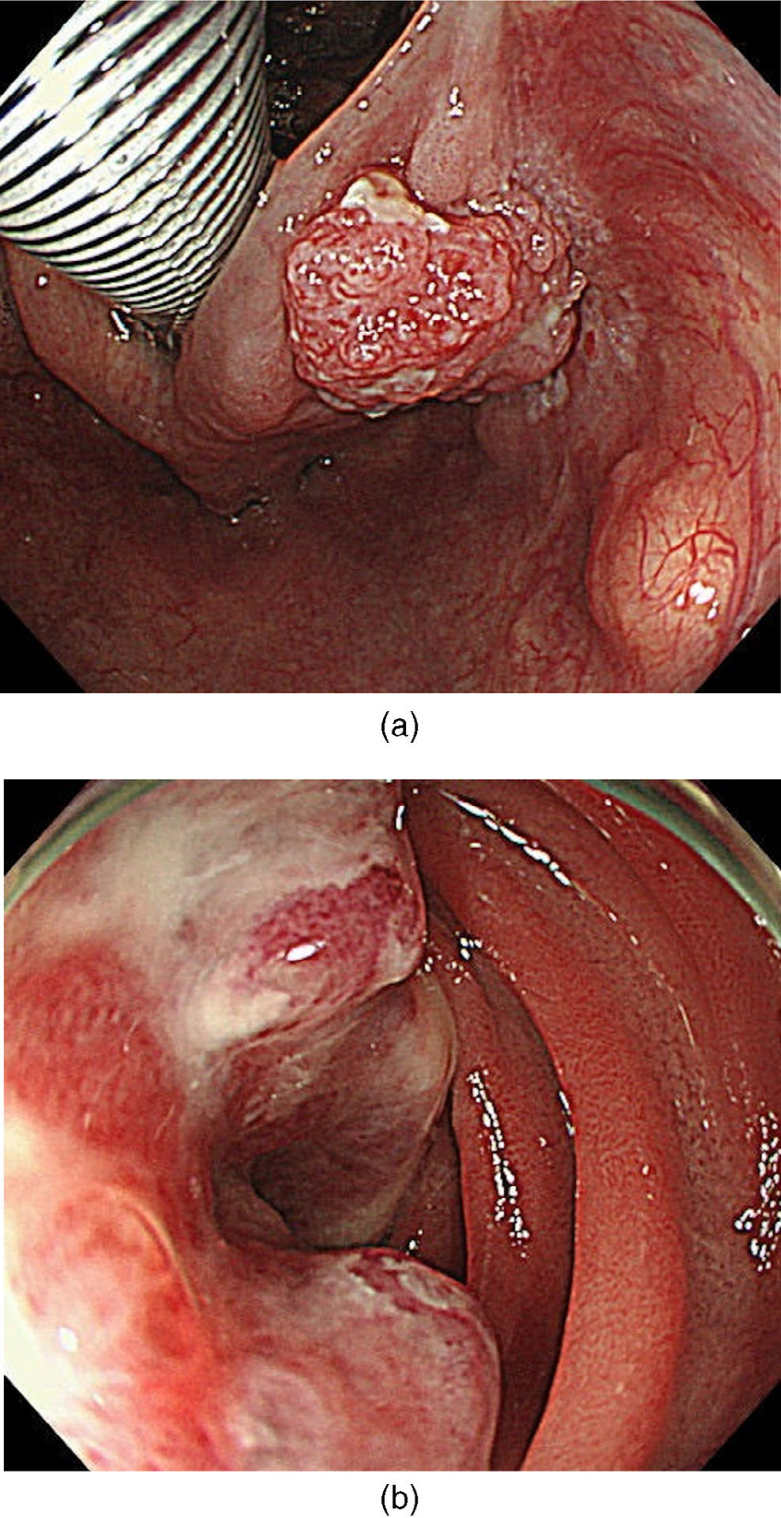
(a) White light imaging. A red protruding tumor located in the pisiform fossa. (b) White light imaging. A lesion of recurrent renal cell carcinoma bumping from the duodenal wall near the papilla of Vater.

Our 69‐year‐old male patient had a history of smoking 20 cigarettes sticks daily and consuming 500 mL of beer daily for the past 40 years. He was diagnosed with RCC at the age of 47 years (22 years ago). Consequently, he underwent a right nephrectomy; however, he was retreated four times, including surgeries for left parotid recurrence (age 52), left arm recurrence (age 53), distal pancreatectomy for pancreatic recurrence (age 55), and heavy particle radiation therapy for remaining pancreatic recurrence (age 64). Three months ago, a follow‐up fluorodeoxyglucose‐positron emission tomography/computed tomography (FDG‐PET/CT) indicated FDG accumulation in the hypopharynx (Figure 1a), duodenum (Figure 1b), liver, and gluteus medius muscle. The patient was diagnosed with squamous cell carcinoma cT2N0M0 of the hypopharynx (Figure 2a) and recurrent RCC of the duodenum (Figure 2b), liver, and gluteus medius muscle, according to hypopharyngeal and liver biopsy.

**FIGURE 3:**
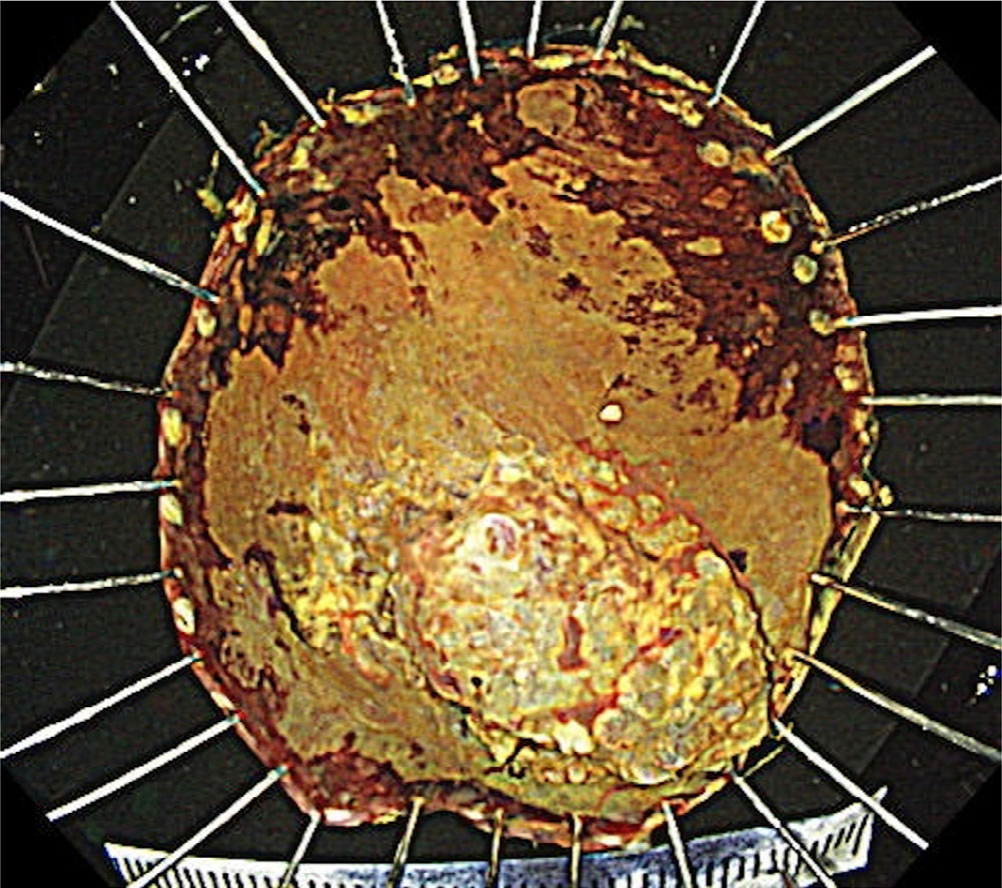
The resected lesion (squamous cell carcinoma, 35 × 32 mm, 0‐Is+IIb, tumor thickness 4000 µm, Ly0, V1, pHMx, and pVM0).

**FIGURE 4:**
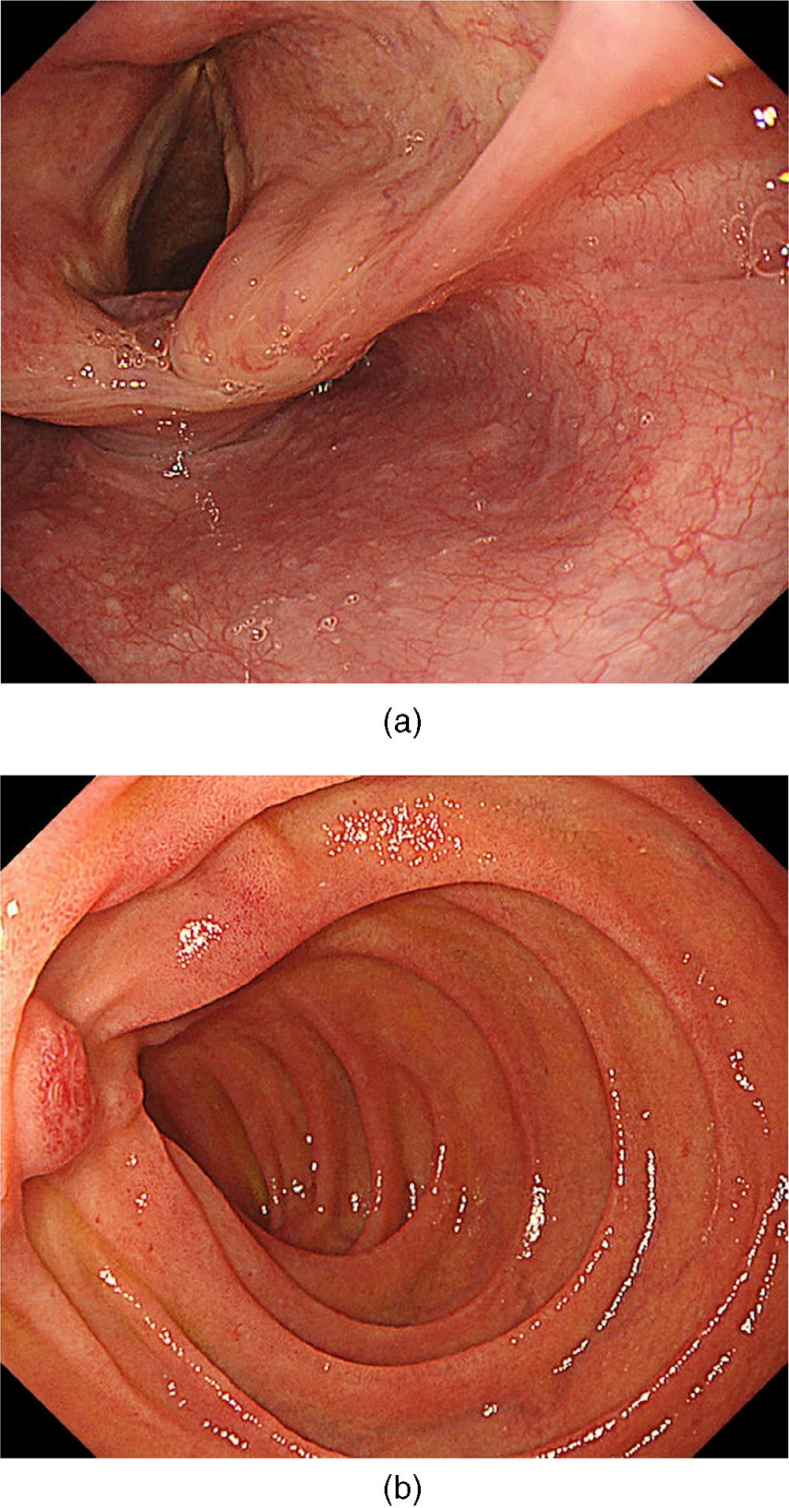
(a) Hypopharyngeal cancer in the pisiform fossa completely resected by endoscopic submucosal dissection. Signs of local recurrence were not noted. (b) The duodenal lesion of recurrent renal cell carcinoma disappeared after the completion of immunotherapy with ipilimumab and nivolumab.

We had to treat two malignancies. Early initiation of immunotherapy was preferable to treat extensively advanced RCC; therefore, we decided to first start immune checkpoint inhibitors (ICIs), ipilimumab and nivolumab, for RCC followed by ESD for local control of hypopharyngeal cancer. According to the IMDC (International Metastatic RCC Database Consortium), the Risk Model for Metastatic RCC, in this case, was considered low to intermediate risk, with a median overall survival of 22.5–43.2 months. The prognosis for RCC is expected to some extent, therefore we determined that there were indications for treatment for pharyngeal cancer. If we wait for the end of treatment of RCC, the hypopharyngeal carcinoma may progress during that time and a more invasive treatment such as surgery may be necessary. Furthermore, total neck irradiation may be necessary for advanced hypopharyngeal cancer. Total neck irradiation partially covers the lungs, and exposure to radiation after the use of ICIs increases the risk of immune‐related adverse events pneumonitis. For these reasons, we decided that it would be better to treat the hypopharyngeal cancer simultaneously. ESD was performed one month after the first course of ipilimumab and nivolumab. After one cycle of ICIs, the lesion was unchanged in size from the previous endoscopy, estimated with iodine staining. We used the technique of peroral countertraction to resect with negative margins. ESD revealed squamous cell carcinoma of 35 × 32 mm, subclassification for superficial cancers of 0–Is + IIb, tumor thickness of 4000 µm, lymphatic invasion of Ly0, venous invasion of V1, proximal margin of pHMx, and vertical margin of pVM0 (Figure 3). No complications such as dysphagia or stenosis occurred. We then performed four courses of ipilimumab and nivolumab and maintenance therapy with nivolumab for RCC. After one year, follow‐up CT revealed no metastasis in the duodenum, liver, or gluteus medius muscle, which was considered a complete response. The patient was regularly followed up by CT and upper gastrointestinal endoscopy (Figure 4a,b) for 3 years, revealing no obvious recurrence of both hypopharyngeal cancer and RCC.

## DISCUSSION

In this case, we chose ESD as the therapy for hypopharyngeal cancer in a double cancer situation. Some reports have indicated the superiority of ESD over endoscopic mucosal resection (EMR) in terms of en bloc and R0 resection rates.^1^ En bloc resection by EMR was difficult in this case because of the complexity of the anatomical structure of the pisiform fossa. Further, endoscopic laryngopharyngeal surgery (ELPS) has been performed for pharyngeal cancer. The superiority between ESD and ELPS remains controversial, but we perform ESD because of our greater experience.

Treatment options for cT2N0M0 hypopharyngeal cancer included chemotherapy, radiation therapy, surgical pharyngectomy, and endoscopic procedures.^2^ We had to treat the hypopharyngeal cancer in parallel with systemic immunotherapy for RCC in this case. However, chemotherapy for hypopharyngeal cancer includes platinum‐based drugs, taxanes, and metabolic antagonists such as 5‐fluorouracil.^3^, ^4^ These drugs are completely different from those used in immunotherapy for RCC. Further, radiation therapy takes approximately 6 weeks to complete.^2^ Therefore, performing it in parallel with immunotherapy for RCC is challenging. Additionally, the 5‐year cause‐specific survival of radiation therapy for early‐stage hypopharyngeal cancer is reported as 74%,^5^ which is inferior to ESD, which is reported as 100%.^6^ Finally, surgical pharyngectomy is highly invasive. Hence, we decided to perform local control of hypopharyngeal cancer by ESD, which is minimally invasive and can be performed in 1 h. We used the technique of peroral countertraction^7^ to keep a layer of dissection just above the muscle layer.

This case was positive for venous infiltration, so additional treatment after ESD was worth considering. However, there is no definite evidence for additional treatment for cases with positive venous invasion after endoscopic resection. It was difficult to perform conventional chemotherapy concurrently with immunotherapy for RCC, therefore we did not provide additional treatment. Further studies are needed on additional treatment for patients with venous or lymphatic invasion after ESD treatment.

Many studies reported on the effectiveness of ESD for local control of superficial pharyngeal cancer and favorable long‐term prognosis postprocedure. In 2015, Kinjo et al. reported 100% and 59% en bloc and R0 resection rates of ESD, respectively.^6^ This study included EMR cases, but the 5‐year overall and cause‐specific survival rates were 80.7% and 100A%, respectively. Katada et al. reported a nationwide multicenter study in Japan on transoral surgery for superficial head and neck cancer.^8^ However, the 3‐year overall, relapse‐free, and cause‐specific survival rates were 88.1%, 84.4%, and 99.6%, respectively.

T2 is defined as tumor invasion into the muscularis propria in the field of gastrointestinal cancer. It is currently defined by the tumor size of 20–40 mm in the field of pharyngeal cancer. However, Ogasawara et al. reported that tumor thickness rather than size correlates with the rate of lymph node metastasis.^9^ Therefore, the staging criteria are expected to be more focused on vertical tumor invasion. Moreover, some studies have investigated the prediction of the invasion depth of superficial pharyngeal cancer by magnifying endoscopy with narrow‐band imaging (ME‐NBI). Kikuchi et al. revealed that the invasion distance was predictable by microvasculature type and categorized by ME‐NBI into B1, B2, and B3.^10^ We demonstrated a B3 microvasculature type in this case. Therefore, we performed ESD for deeper resection, resulting in VM0 resection.

In conclusion, ESD is less invasive and can be performed in a shorter time than other treatment options for superficial hypopharyngeal cancer. It is effective in controlling local lesions, causing a tolerable prognosis. Herein, we report a case of ESD for hypopharyngeal cancer performed in parallel with systemic therapy for other malignancies. ESD may be particularly suited for patients with pharyngeal cancer combined with other malignancies.

## CONFLICT OF INTEREST STATEMENT

None.

## ETHICS STATEMENT

Approval of the research protocol by an Institutional Reviewer Board: N/A.

## PATIENT CONSENT STATEMENT

Informed consent was obtained from the patient.
